# Comment on response from Milesi et al. to 'Perinatal exposure to a glyphosate-based herbicide impairs female reproductive outcomes and induces second-generation adverse effects in Wistar rats'

**DOI:** 10.1007/s00204-019-02647-8

**Published:** 2019-12-18

**Authors:** Ian Plewis

**Affiliations:** University of Manchester, Manchester, UK

Dear Editors,

Milesi et al. (2018, 2019) are to be applauded for their willingness to reanalyse the data used in their 2018 paper to take account of litter effects in their three-generation glyphosate study. As proposed by Plewis (2019), they now use appropriate statistical models (linear mixed models, often known as multilevel or hierarchical linear models) to take account of dependence between offspring from the same dam. This approach is more efficient than using litter as the unit of analysis as suggested by Paumgartten (2019) and others. Note that litter effects are just one way in which observational dependence is generated in rodent feeding experiments; cage effects and repeated measures are others. Unfortunately, the reanalyses of Milesi et al. (2019) do not go far enough in terms of allowing for the hierarchical structure of their data and assertions that their original findings hold up after the reanalyses are not borne out.

Consider first the model that lies behind Table 1 and using algebra rather than the confusing R syntax employed by the authors:

$$ y_{ij} = \beta_{0} + \mathop \sum \limits_{l = 1}^{2} \beta_{l} t_{lj} + u_{j} + e_{ij} , $$

where $y_{ij}$ is the pre-implantation loss % for each F1 rat; $i = 1 \ldots n_{j}$ ( max $n_{j}$ = 4) indexes the F1 rats within F0 dams $j,\;\;j = 1 \ldots 21$; $t_{lj}$ are dummy (0/1) fixed effects for the LD and HD treatments; $u_{j} \sim N\left( {0,\sigma_{{\text{u}}}^{2} } \right)$ is the random effect for F0 with $\sigma_{{\text{u}}}^{2}$ being the variance between F0 dams and $e_{ij} \sim N\left( {0,\sigma_{{\text{e}}}^{2} } \right),$ where $\sigma_{{\text{e}}}^{2}$ is the variance between F1 rats within the F0 dams.

The null hypothesis is that $\beta_{1} = \beta_{2} = 0$ and this should be tested using a single Wald test rather than using two separate tests as Milesi et al. (2019) do. Nevertheless, they do not find a statistically significant difference between the LD group and the controls and this is out of line with Fig. 3d (Milesi et al. 2018, p. 2635).

There are a number of points to note about this model and the data used by Milesi et al. (2019) to estimate its parameters. First is that the treatments vary only between F0 dams; all F1 offsprings are exposed to the same treatment. Second, the litter effect can be represented as the proportion of the total variance in the outcome explained by the F0 dams, i.e. $\sigma_{{\text{u}}}^{2} /\left( {\sigma_{{\text{u}}}^{2} + \sigma_{{\text{e}}}^{2} } \right)$, often known as the intra-cluster coefficient. Milesi et al. (2019) do not provide this estimate which is regrettable, because it would be a helpful indicator for the design of future studies even though a precise estimate of $\sigma_{{\text{u}}}^{2}$ might be difficult to obtain with such a small sample (21) of F0 dams. Third, there seems to be unexplained missing data as we would expect a F1 sample size of 28 for each of the three conditions, rather than 25, 20 and 20 as given and these numbers fall further at F2 to 20, 15 and 13. One implication of the way the F1 rats were apparently housed in cages (four per cage; Milesi et al. 2018, p. 2631) is that the variation in the outcome between F0 dams includes both a genetic effect and an environmental (or cage) effect arising from the co-housing. Ideally, these two effects should be separated by, for example, housing only two F1 rats together. Finally, the outcome variable, $y_{ij}$, is a percentage which might be very small for many F1 rats, suggesting that rather than assuming normality for $e_{ij}$, a binomial model might be more appropriate.

Turning to Table 2, we find that the authors do not fit the three-level models (F2 nested within F1 nested within F0) that are required, i.e.,

$$ y_{ijk} = \beta_{0} + \mathop \sum \limits_{l = 1}^{2} \beta_{l} t_{lk} + v_{k} + u_{jk} + e_{ijk} , $$

where $y_{ijk}$ is now the outcome of interest (fetal weight etc.) for each F2 rat; $i = 1 \ldots n_{jk}$ indexes the F2 rats within F1 $j,\;\;j = 1 \ldots J_{k}$ and F0 $k,\;\;k = 1 \ldots K$; $t_{lk}$ are the treatment variables as before; $v_{k} \sim N\left( {0,\sigma_{{\text{v}}}^{2} } \right)$ is now the random effect for F0 dams; $u_{jk} \sim N\left( {0,\sigma_{{\text{u}}}^{2} } \right)$ is now the random effect for F1 (within F0) and $e_{ijk} \sim N\left( {0,\sigma_{{\text{e}}}^{2} } \right)$ with $\sigma_{{\text{e}}}^{2}$ the variance between F2 rats within both the F0 and F1 dams. Models such as these are easily estimated with the appropriate software and estimates of the variance components can inform future designs.

Even though Milesi et al. (2019) wrongly omit the F0 level from the model underpinning Table 2, their results are still different from those reported in the original paper: there is no longer any treatment effect for placental weight or for foetal length for the low dose. It is plausible to suppose that allowing for the full hierarchical structure would lead to a further reduction in the precision of the estimated treatment effects.

The authors are of course correct to state that no one experiment can be definitive. Arguably, more attention to experimental design is warranted to have appropriately powered multi-generational rodent experiments such as this one and others (e.g. Kubsad et al. 2019). But rather than offer their rats for further experiments, it would be much more helpful to researchers interested in estimating litter effects, if they made their data publicly available.
